# Enzymatic Debridement in Geriatric Burn Patients—A Reliable Option for Selective Eschar Removal

**DOI:** 10.3390/jcm12072633

**Published:** 2023-03-31

**Authors:** Christian Tapking, Victoria G. Rontoyanni, Yannick F. Diehm, Felix Strübing, Farzan Solimani, Amir K. Bigdeli, Gabriel Hundeshagen, Sebastian Fischer, Ulrich Kneser, Laura C. Siegwart

**Affiliations:** 1 Department of Hand, Plastic and Reconstructive Surgery, Microsurgery, Burn Center, BG Klinik Ludwigshafen, University of Heidelberg, 67071 Ludwigshafen, Germany; 2 Department of Surgery, University of Texas Medical Branch, Galveston, TX 77555, USA; 3 Department of Dermatology, Venereology and Allergology, Charité Universitätsmedizin Berlin, Humboldt-Universität zu Berlin, 10099 Berlin, Germany; 4 Berlin Institute of Health at Charité, Universitätsmedizin Berlin, BIH Biomedical Innovation Academy, BIH Charité Clinician Scientist Program, 10178 Berlin, Germany

**Keywords:** burns, surgery, elderly, enzymatic debridement, NexoBrid©

## Abstract

The treatment of geriatric burn patients represents a major challenge in burn care. The objective of this study was to evaluate the efficacy of enzymatic debridement (ED) in geriatric burn patients. Adult patients who received ED for treatment of mixed pattern and full thickness burns (August 2017–October 2022) were included in this study and grouped in the younger (18–65 years) and geriatric (≥65 years) groups. Primary outcome was a necessity of surgery subsequent to ED. Both groups (patient characteristics, surgical and non-surgical treatment) were compared. Multiple logistic and linear regression models were used to identify the effect of age on the outcomes. A total of 169 patients were included (younger group: 135 patients, geriatric group: 34 patients). The burn size as indicated by %TBSA (24.2 ± 20.4% vs. 26.8 ± 17.1%, *p* = 0.499) was similar in both groups. The ASA (2.5 ± 1.1 vs. 3.4 ± 1.1, *p* < 0.001) and ABSI scores (6.1 ± 2.8 vs. 8.6 ± 2.3, *p* < 0.001) were significantly higher in the geriatric group. The %TBSA treated with ED (5.4 ± 5.0% vs. 4.4 ± 4.3%, *p* = 0.245) were similar in both groups. The necessity of additional surgical interventions (63.0 % vs. 58.8 %, *p* = 0.763) and the wound size debrided and grafted (2.9 ± 3.5% vs. 2.2 ± 2.1%; *p* = 0.301) were similar in both groups. Regression models yielded that age did not have an effect on efficacy of ED. We showed that ED is reliable and safe to use in geriatric patients. Age did not have a significant influence on the surgical outcomes of ED. In both groups, the size of the grafted area was reduced and, in many patients, surgery was avoided completely.

## 1. Introduction

According to the World Health Organization, there is an ongoing demographic change and the geriatric population (>60 years) will have increased from 12% to 22% between 2015 and 2022, leading to major challenges for the health care and social systems [1]. Geriatric patients are susceptible to trauma and burn injuries in particular, due to limited vision and mobility, decreased physical strength, and coordination resulting in slow reactions to dangerous situations [2,3,4]. In this context, the percentage of geriatric patients with need for burn care is expected to increase in the future.

Despite substantial advances in both surgical burn treatment and intensive care, age remains a significant predictor for mortality in burn patients [5]. The literature shows that survival rates in geriatric patients have increased in the last decades, but have still not reached those of younger patients [6,7,8]. Early excision of burn eschar has become a major principle in the acute treatment of severe burns [9,10]. Conventionally, burn eschar was surgically removed. Another valuable option is the use of enzymatic debridement (ED) (NexoBrid^®^, Mediwound GmbH, Rüsselsheim, Germany) [11,12]. The agent, a Bromelain-based enzyme complex, selectively dissolves the burn eschar without harming healthy tissue. This unique mechanism preserves the potential for spontaneous re-epithelialization out of viable dermis remnants. ED was proofed to be efficient in partial and full thickness burn wounds, in particular in critical body parts with delicate skin such as the hands and face [13,14]. There are some studies showing that ED might even lead to aesthetically and functionally better outcomes compared to conventional surgical interventions [14,15,16]. ED is also effective at preventing burn-induced compartment syndrome in circumferential burns, avoiding surgical escharotomy [17,18]. However, the number and quality of studies on ED in the literature is limited. Most articles present retrospective chart reviews and few prospective randomized controlled studies exist [19]. It is evident that the human skin becomes fragile with age [20]. To be precise, the skin becomes thin, vascularity and cellularity are reduced, and there is a loss and disorganization of collagen fibers, decreasing the resistance to external agents and injuries [21].

Therefore, the primary objective of this study was to evaluate the efficacy of ED in geriatric burn patients (≥65 years). We compared the use of ED in the younger to the geriatric patient group and identified variables that influence successful ED, meaning, here, the absence of additional surgical intervention.

## 2. Materials and Methods

This retrospective study was approved by the Ethics Commitee Rheinland-Pfalz previous to initiating the study (2021-15809). The study population included all patients admitted to our burn unit between August 2017 and October 2022 who received NexoBrid^®^ (Mediwound GmbH, Rüsselsheim, Germany) for ED. Age at burn was the primary risk factor in binary form. Patients were classified into a younger group (18–65 years) and a geriatric group (≥65 years).

### 2.1. Enzymatic Debridement

The primary indication for ED were mixed, partial, or full thickness scald or flame burns on the hands and lower arms. ED was applied to a maximum of 15% TBSA in adult patients (≥18 years). The exclusion criteria for ED application were chemical burns, evident compartment syndrome, and an age <18 years. The indication for ED was validated by experienced burn surgeons (>50 ED applications). ED was performed as reported in previous studies from our institution [17,22]. The indications for ED and product application followed both the manufacturer guidelines and the European consensus [23,24].

Subsequent to sufficient ED of at least 4 hours, the wound bed was evaluated regarding color and bleeding patterns [25]. At this point, the decision of whether the wounds were to be treated conservatively or require additional surgical debridement and grafting was made.

### 2.2. Outcome Measures

The primary outcome was the necessity of surgery after ED. Further information data on surgical and non-surgical treatment such as time to ED, % total body surface area (TBSA) for ED, time to surgery, TBSA for surgery, and length of hospital stay were collected (see Table 1 and Table 2).

### 2.3. Patient Demographics and Clinical Characteristics

Demographic data (age, sex) and injury data (% TBSA, presence of inhalation injury), the ASA-Score (American Society of Anesthesiologists) and the ABSI-Score (Abbreviated Burn Severity Index) were collected. All data were extracted from electronic medical charts.

### 2.4. Statistical Analysis

Descriptive statistics are presented as group mean ± SD, or as frequencies and proportions, as appropriate. Patient characteristics and clinical outcomes were tested for group mean differences using unpaired two-tailed Student *t*-tests and for differences in proportions using chi-squared tests.

Multiple logistic regression analysis was used to assess the association between age group and whether surgical intervention followed the ED. Both forward selection and backward elimination was employed based on a significance level at a = 0.2 and Akaike’s Information Criterion (AIC), resulting in the same final regression model. Model diagnostics for outliers and collinearity were performed on the final regression model.

To assess the relationship between age and burn size (% TBSA) that was surgically treated subsequent to initial ED, we used multiple linear regression analysis, adjusting for patient and injury characteristics identified via a forward model selection process based on a significance level at a = 0.2. In this analysis, only patients who received both treatments were included. Model diagnostics for model assumptions, outliers, and collinearity were examined in the final model. A *p* value of ≤0.05 was considered statistically significant. All analyses were performed in SAS (Version 9.4; SAS Institute Inc., Cary, NC, USA).

## 3. Results

### 3.1. Patient Characteristics

A total of 169 patients (141 males, 28 females) with 143 flame burns and 26 scald burns were included in the study. A total of 135 patients were included in the younger group and 34 patients were included in the geriatric group.

The two groups significantly differed in mean age (39.6 ± 13.7 years vs. 77.4 ± 7.3 years, *p* < 0.001). The burn size, indicated by %TBSA (24.2 ± 20.4% vs. 26.8 ± 17.1%, *p* = 0.499), was similar in both groups.

The ASA (2.5 ± 1.1 vs. 3.4 ± 1.1, *p* < 0.001) and ABSI scores (6.1 ± 2.8 vs. 8.6 ± 2.3, *p* < 0.001) were significantly higher in the geriatric group. Additionally, in the geriatric group, more patients presented with inhalation injury (25.2% vs. 50.0%, *p* = 0.005) and needed catecholamines (17.0% vs. 47.1%, *p* = 0.002) at admission.

### 3.2. Treatment Characteristics

The %TBSA treated with ED (5.4 ± 5.0 %vs. 4.4 ± 4.3 %, *p* = 0.245) was similar between both age groups. There was no significant difference in the time from injury to ED (0.6 ± 0.9 days vs. 0.9 ± 1.8 days, *p* = 0.246) and the time from ED to surgery (9.1 ± 7.2 days vs. 5.9 ± 2.9 days, *p* = 0.053) between the age groups.

Patient characteristics and treatment characteristics are presented by age group (18–65 years vs. ≥65 years) in Table 1.

### 3.3. Clinical Outcomes

There was no significant difference in the necessity of additional surgical intervention between both age groups (63.0% vs. 58.8%, *p* = 0.763). The burn size that was surgically debrided and grafted subsequent to ED was not significantly different between the age groups (2.9 ± 3.5% vs. 2.2 ± 2.1%; *p* = 0.301), and neither was the length of the hospital stay (29.4 ± 23.8 days versus 30.3 ± 29.0 days, *p* = 0.805). The mortality rate was significantly lower in the younger group (8.9% vs. 47.1%, *p* ≤ 0.001). In none of the patients of both groups, adverse events (allergic reactions, excessive pain, damage to healthy skin) occurred.

Treatment-related outcomes are shown in Table 2.

### 3.4. Model Selection and Regression Estimates

Using multiple logistic regression, we identified predictive variables for the necessity of additional surgical intervention subsequent to ED. The final regression model after a variable selection process included burn size treated with ED, type of burn, and sex, while age group was forced in the model. A larger area that was treated with enzymatic debridement and female sex were positive predictors. The estimated odds of having surgery as a female are 3.03 [95% Wald CI: 1.12, 8.19] times the odds of having surgery as a male, after adjusting for TBSA that was debrided with ED, age group, and the type of burn. For one unit increase in TBSA that was treated with ED, the estimated odds of having more surgery are 1.145 [95% Wald CI: 1.042, 1.258] times greater, after adjusting for age, type of burn, and sex. There was no influence of type of burn (flame vs. scald) or age group. The results of the multiple logistic regression analysis are shown in Table 3.

Using multiple linear regression, the predictors for the size of the wound, which was initially debrided enzymatically and then surgically, were identified. The model yielded that the surgically treated area was larger when the enzymatically debrided area was larger, inhalation injury was present, the overall burn size was larger, and the application time longer. There was no influence of age, respectively. The results of the multiple linear regression analysis are shown in Table 4.

## 4. Discussion

This is the first study to evaluate the efficacy of ED in geriatric burn patients. The efficacy of ED was defined as the necessity of surgical intervention subsequent to ED as well as the reduction of the size of the burn wound that required surgical debridement and grafting. The present study found no significant influence of age on the necessity of surgical intervention in burn wounds first debrided with ED. Age was also no predictive factor for wound size that required surgical debridement and grafting.

Up until now, it is elusive whether age impacts successful ED. Aging is associated with substantial changes of the skin anatomy that diminish integrity and function and reduce its healing potential [26]. Beginning at about 60 years of age, the skin becomes thinner at the dermo-epidermal interface because dermal papillae between the skin layers decrease. This process is associated with reduced vascularity and cellularity of the skin, contributing to low tissue oxygenation and changes of the ground substance, including loss and disorganization of collagen fibers [26]. ED is selective to collagen [27]. In detail, the active enzyme complex involves multiple collagenases that selectively dissolve thermally damaged collagen fibers. Those will subsequently be washed out by wound clearing. This study leads to the assumption that ED can be used safely in any adult patient and that the indications for its use do not need to vary among younger and geriatric burn patients. It was shown that the wound size that needed additional surgical intervention was reduced in both age groups. In the present study, this parameter does not provide evidence on ED efficacy, since the wound size could have decreased by spontaneous healing. However, it disproves the fear that ED could harm the vulnerable healthy skin in geriatric patients and that the diminished healing potential compromises the advantages of non-invasive eschar removal due to the need for additional skin grafting.

Linear regression models in this study showed that the initial burn size, the wound size treated with ED, and the ED application time were significant predictors for the wound size that needed additional surgical intervention. Age did not have a significant influence. Overall, the mean wound size that needed to be surgically treated was smaller than the wound size initially treated with ED. In line with this, Cordts et al. outlined that the skin-grafted areas of burn wounds treated with ED could be reduced by 37% compared to the initial assessments [13]. Therefore, one might argue that the efficacy of ED is not only present when additional surgical intervention is avoided, but also when the size of the wound that needs skin grafting is reduced or when optimal wound bed preparation is accomplished through selective and complete eschar removal. However, to evaluate the efficacy of ED, a comparison to other treatment modalities in control is needed. In addition, ED can also be used to prevent surgical escharotomy, not primarily aiming to reduce skin grafting [17].

The differences between the groups included in this study regarding ASA- and ABSI-Scores as well as mortality can mainly be explained by the age. Firstly, patient age is a main contributor to the above-mentioned scores. Secondly, numberous previously published studies demonstrated the major effect of advanced age on mortality rates in burns. It has been reported that children have the best outcome, followed by adults and the elderly, with only minor improvements in recent years [5,28]. Jeschke et al. showed that the LD50 decreases from 50% TBSA to 25% TBSA from the age of 55 years to 70 years [7].

Similar to higher ASA- and ABSI-Scores in the geriatric group, those patients also more frequently needed catecholamines during their unstable circulatory state at admission. The use of catecholamines may lead to disturbances in the burn wounds, but also in the wound assessment, since vasopressors reduce the microperfusion of the skin, including chronic and burn wounds [29]. This is in line with our experience that the evaluation of enzymatically debrided burn wounds is more challenging in geriatric patients, due to the thinned skin layers and reluctant wound bleeding. Of note, clinical criteria such as pattern and dynamics of wound bleeding and wound bed color are key criteria for the decision to conduct surgical or conservative therapy subsequent to ED [26].

In the present study, the logistic regression showed that the burn mechanism (scald vs. flame) was not a predictive factor of necessity of additional surgical debridement. This is contrary to a study that was previously published by our group that investigated the efficacy of ED in scald versus flame burns. It was found that scald burns needed to be debrided surgically after ED far more often [23]. This phenomenon was also reported by Kwa et al., who figured that many scalds do not denature collagen properly leading to ED not being able to remove the eschar [27,30]. The difference between the two studies may be due to the other statistical approach and/or the larger sample size in the present study.

In our burn center, the percentage of patients who needed additional surgical intervention was rather high––around 60% in both groups. Similar percentages of surgical debridement and grafting subsequent to ED were reported by Dadras et al., while Rivas-Nicolls et al. reported additional surgical intervention in 38% of the patients [31,32]. However, we follow a clear policy in which we take decisions early and pursue definitive treatments as soon as possible in order to reduce the hospital stay, rate of infections, and immobilization.

The length of stay following burn injury is usually estimated at 1 day per %TBSA, but is often exceeded as outlined by Taylor et al. [33]. In accordance with this, both groups slightly exceed the length of stay over the %TBSA. Surprisingly, the length of stay was similar in the younger and the geriatric group. This may be due to age and comorbidities in the geriatric group. In our burn unit, many of the patients in the younger group were admitted due to work-related burn injuries. Those patients profit from the employer’s liability insurance coverage and stay in our burn unit until admission to the rehabilitation facilities.

### Limitations

The limitations of this study should be considered. This is a retrospective single center analysis based on a prospectively maintained data base, including all patients who received ED in our burn center. In contrast to prospective studies, no information on drop-out patients were collected. Even though the number of patients included in the present study is rather low, this is one of the largest studies on ED that has been published in the literature. One could assume that ED was more liberally applied in geriatric patients in order to avoid surgery and associated risks in this patient group, which was more unstable at admission. However, there was no significant influence of age on the mentioned outcomes. Other institutions may have other experiences and approaches using ED in geriatric burn patients. Therefore, multicenter studies are crucial and should be performed in future to verify the presented results. In our institution, burn depth is usually assessed clinically without objective measures, such as laser doppler imaging, which may support finding the proper patients and wounds for ED. This may have influenced the rate of surgical intervention and autografting.

When evaluating the efficacy of ED, it is crucial, that all patients were treated similarly regarding the indication of ED and additional surgical intervention as well as standardized ED application. In this context, it is important to mention that experienced burn surgeons need to be involved in the process of ED (assessment of the burn wound previous and subsequent to ED, application of ED, indication for ED, and additional surgical intervention).

## 5. Conclusions

The present study is the first report on the efficacy of enzymatic debridement in geriatric burn patients. We showed that age did not have a significant influence on the surgical outcomes of ED. The product can be used safely and works reliably in geriatric patients. In both the younger and the geriatric patient group, the size of the grafted area was reduced and, in many patients, surgery was avoided completely. We believe that additional multicenter studies may help to find the “ideal cohort” that profits the most from ED. Independent from treatment with ED, more studies on how to improve the outcome of geriatric burn patients are needed in order to decrease the mortality in this vulnerable patient collective.

## Figures and Tables

**Table 1:** Patient demographics and treatment characteristics.

	Younger N = 135	Geriatric N = 34	*p*-Value
Females, n (%)	20 (14.8)	8 (23.5)	0.222
Age at burn (years)	39.6 ± 13.7	77.4 ± 7.3	<0.001
%TBSA	24.2 ± 20.4	26.8 ± 17.1	0.499
Type of burn (flame), n (%)	114 (84.4)	29 (85.3)	0.902
BMI	27.1 ± 6.7	26.6 ± 3.8	0.670
ASA score	2.5 ± 1.1	3.4 ± 1.1	<0.001 ^#^
ABSI score	6.1 ± 2.8	8.6 ± 2.3	<0.001 ^#^
Inhalation injury, n (%)	34 (25.2)	17 (50.0)	0.005 ^#^
Catecholamines at admission, n (%)	23 (17.0)	16 (47.1)	0.002 ^#^
%TBSA treated ED *	5.4 ± 5.0	4.4 ± 4.3	0.245
Time from injury to ED (days)	0.6 ± 0.9	0.9 ± 1.8	0.246
Time from ED to surgery (days)	9.1 ± 7.2	5.9 ± 2.9	0.053
Time of ED application (minutes)	265.9 ± 65.8	260.6 ± 67.9	0.780

Data is displayed as mean ± SD or n (%), as applicable. TBSA—total body surface area, *—%TBSA that was treated with ED, BMI—Body Mass Index, ASA—American Society of Anesthesiologists, ED—enzymatic debridement, ^#^ *p* < 0.05.

**Table 2:** Clinical outcomes.

	Younger N = 135	Geriatric N = 34	*p*-Value
Mortality, n (%)	12 (8.9)	16 (47.1)	<0.001 ^#^
Patients with additional surgical intervention, n (%) *	85 (63.0)	20 (58.8)	0.763
%TBSA surgically treated **	2.9 ± 3.5	2.2 ± 2.1	0.301
LOS	29.4 ± 23.8	30.3 ± 29.0	0.805

Data is displayed as mean ± SD or n (%), as applicable. TBSA—total body surface area, LOS—length of hospital stay, *—number of patients with surgery in same location as enzymatic debridement, **—%TBSA treated in location that was treated with enzymatic debridement before, ^#^ *p* < 0.05.

**Table 3:** Logistic regression predicting necessity of additional surgical intervention (n = 167).

Effect	Point Estimate, OR	95% Wald Confidence Levels		*p*-Value
%TBSA treated ED	1.145	1.042	1.258	0.005 ^#^
Type of burn (scald)	2.727	0.943	7.887	0.064
Sex (female)	3.032	1.123	8.188	0.029 ^#^
Age group (elderly)	0.969	0.420	2.238	0.941

TBSA—total body surface area, ED—enzymatic debridement, ^#^ *p* < 0.05.

**Table 4:** Linear regression predicting the burn size debrided enzymatically versus surgically (n = 167).

Effect	Estimate	Standard Error	*p*-Value
%TBSA treated ED	0.275	0.049	<0.001 ^#^
Time of ED application (min)	0.008	0.008	0.043 ^#^
TBSA	0.057	0.057	0.001 ^#^
Age group (elderly)	0.010	0.603	0.987
Inhalation injury	−1.394	0.598	0.021 ^#^

TBSA—total body surface area, min—minutes, ED—enzymatic debridement, ^#^ *p* < 0.05.

## Data Availability

The data presented in this study are available on request from the corresponding author. The data are not publicly available due to ethical, legal and privacy issues.

## References

[1] World Health Organization (2022). Aging and Heath Fact Sheet.

[2] Gioffrè-Florio M., Murabito L.M., Visalli C., Pergolizzi F.P., Famà F. (2018). Trauma in elderly patients: A study of prevalence, comorbidities and gender differences. Il G. Di Chir..

[3] Jeschke M.G., Patsouris D., Stanojcic M., Abdullahi A., Rehou S., Pinto R., Chen P., Burnett M., Amini-Nik S. (2015). Pathophysiologic Response to Burns in the Elderly. eBioMedicine.

[4] Abu-Sittah G., Chahine F.M., Janom H. (2016). Management of burns in the elderly. Ann. Burn. Fire Disasters.

[5] Wearn C., Hardwicke J., Kitsios A., Siddons V., Nightingale P., Moiemen N. (2015). Outcomes of burns in the elderly: Revised estimates from the Birmingham Burn Centre. Burns.

[6] Lionelli G.T., Pickus E.J., Beckum O.K., Decoursey R.L., Korentager R.A. (2005). A three decade analysis of factors affecting burn mortality in the elderly. Burns.

[7] Jeschke M.G., Pinto R., Costford S.R., Amini-Nik S. (2016). Threshold age and burn size associated with poor outcomes in the elderly after burn injury. Burns.

[8] Macrino S., Slater H., Aballay A., Goldfarb I.W., Caushaj P.F. (2008). A three-decade review of thermal injuries among the elderly at a regional burn centre. Burns.

[9] Herndon D.N., Barrow R.E., Rutan R.L., Rutan T.C., Desai M.H., Abston S. (1989). A comparison of conservative versus early excision. Therapies in severely burned patients. Ann. Surg..

[10] Wu X.-W., Herndon D.N., Spies M., Sanford A.P., Wolf S.E. (2002). Effects of delayed wound excision and grafting in severely burned children. Arch. Surg..

[11] Klasen H.J. (2000). A review on the nonoperative removal of necrotic tissue from burn wounds. Burns.

[12] Rosenberg L., Lapid O., Bogdanov-Berezovsky A., Glesinger R., Krieger Y., Silberstein E., Sagi A., Judkins K., Singer A.J. (2004). Safety and efficacy of a proteolytic enzyme for enzymatic burn debridement: A preliminary report. Burns.

[13] Cordts T., Horter J., Vogelpohl J., Kremer T., Kneser U., Hernekamp J.F. (2016). Enzymatic debridement for the treatment of severely burned upper extremities—Early single center experiences. BMC Dermatol..

[14] Schulz A., Fuchs P.C., Rothermundt I., Hoffmann A., Rosenberg L., Shoham Y., Oberländer H., Schiefer J. (2017). Enzymatic debridement of deeply burned faces: Healing and early scarring based on tissue preservation compared to traditional surgical debridement. Burns.

[15] Corrales-Benítez C., González-Peinado D., González-Miranda Á., Martínez-Méndez J.R. (2022). Evaluation of burned hand function after enzymatic debridement. J. Plast. Reconstr. Aesthet. Surg..

[16] Schulz A., Shoham Y., Rosenberg L., Rothermund I., Perbix W., Christian Fuchs P., Lipensky A., Schiefer J.L. (2017). Enzymatic Versus Traditional Surgical Debridement of Severely Burned Hands: A Comparison of Selectivity, Efficacy, Healing Time, and Three-Month Scar Quality. J. Burn Care Res..

[17] Fischer S., Haug V., Diehm Y., Rhodius P., Cordts T., Schmidt V.J., Kotsougiani D., Horter J., Kneser U., Hirche C. (2019). Feasibility and safety of enzymatic debridement for the prevention of operative escharotomy in circumferential deep burns of the distal upper extremity. Surgery.

[18] Grünherz L., Michienzi R., Schaller C., Rittirsch D., Uyulmaz S., Kim B.S., Giovanoli P., Lindenblatt N. (2022). Enzymatic debridement for circumferential deep burns: The role of surgical escharotomy. Burns.

[19] Rosenberg L., Krieger Y., Bogdanov-Berezovski A., Silberstein E., Shoham Y., Singer A.J. (2014). A novel rapid and selective enzymatic debridement agent for burn wound management: A multi-center RCT. Burns.

[20] Dyer J.M., Miller R.A. (2018). Chronic Skin Fragility of Aging. J. Clin. Aesthet. Dermatol..

[21] Jeschke M.G., Pinto R., Kraft R., Nathens A.B., Finnerty C.C., Gamelli R.L., Gibran N.S., Klein M.B., Arnoldo B.D., Tompkins R.G. (2015). Morbidity and survival probability in burn patients in modern burn care. Crit. Care Med..

[22] Tapking C., Siegwart L.C., Jost Y., Hundeshagen G., Kotsougiani-Fischer D., Gazyakan E., Bliesener B., Kneser U., Fischer S. (2022). Enzymatic debridement in scalds is not as effective as in flame burns regarding additional eschar excision: A retrospective matched-control study. Burns.

[23] Hirche C., Almeland S.K., Dheansa B., Fuchs P., Governa M., Hoeksema H., Korzeniowski T., Lumenta D.B., Marinescu S., Martinez-Mendez J.R. (2020). Eschar removal by bromelain based enzymatic debridement (Nexobrid^®^) in burns: European consensus guidelines update. Burns.

[24] Hirche C., Citterio A., Hoeksema H., Koller J., Lehner M., Martinez J.R., Monstrey S., Murray A., Plock J.A., Sander F. (2017). Eschar removal by bromelain based enzymatic debridement (Nexobrid^®^) in burns: An European consensus. Burns.

[25] Siegwart L.C., Böcker A.H., Diehm Y.F., Kotsougiani-Fischer D., Erdmann S., Ziegler B., Kneser U., Hirche C., Fischer S. (2021). Enzymatic Debridement for Burn Wound Care: Interrater Reliability and Impact of Experience in Post-intervention Therapy Decision. J. Burn Care Res..

[26] Romanowski K.S., Sen S. (2022). Wound healing in older adults with severe burns: Clinical treatment considerations and challenges. Burns.

[27] Kwa K.A.A., van Haasterecht L., Elgersma A., Breederveld R.S., Groot M.L., van Zuijlen P.P., Boekema B.K. (2020). Effective enzymatic debridement of burn wounds depends on the denaturation status of collagen. Wound Repair Regen..

[28] Pereira C.T., Barrow R.E., Sterns A.M., Hawkins H.K., Kimbrough C.W., Jeschke M.G., Lee J.O., Sanford A.P., Herndon D.N. (2006). Age-dependent differences in survival after severe burns: A unicentric review of 1674 patients and 179 autopsies over 15 years. J. Am. Coll Surg..

[29] Chakroborty D., Goswami S., Basu S., Sarkar C. (2020). Catecholamines in the regulation of angiogenesis in cutaneous wound healing. FASEB J..

[30] Ali A., Herndon D.N., Mamachen A., Hasan S., Andersen C.R., Grogans R.J., Brewer J.L., Lee J.O., Heffernan J., Suman O.E. (2015). Propranolol attenuates hemorrhage and accelerates wound healing in severely burned adults. Crit. Care.

[31] Rivas-Nicolls D., Aguilera-Sáez J., Gallardo-Calero I., Serracanta J., Gomez P., Palao R., Barret J.P. (2020). Does Enzymatic Debridement Allow Us to Perform Conservative Treatment on Clinically Deep Hand Burns? A Retrospective Review. Ann. Burns Fire Disasters.

[32] Dadras M., Wagner J.M., Wallner C., Sogorski A., Sacher M., Harati K., Lehnhardt M., Behr B. (2020). Enzymatic debridement of hands with deep burns: A single center experience in the treatment of 52 hands. J. Plast. Surg. Hand Surg..

[33] Taylor S.L., Sen S., Greenhalgh D.G., Lawless M., Curri T., Palmieri T.L. (2016). Real-Time Prediction for Burn Length of Stay Via Median Residual Hospital Length of Stay Methodology. J. Burn Care Res..

